# Neuroimmune–Lipid Crosstalk in Chronic Itch: Molecular Mechanisms from Skin Surface to Cerebral Cortex

**DOI:** 10.3390/cells15201891

**Published:** 2026-10-16

**Authors:** Han Bi Kim, Ji Young Um, Da Eun Song, Bo Young Chung, Chun Wook Park, Hye One Kim

**Affiliations:** Department of Dermatology, College of Medicine, Hallym University, Kangnam Sacred Heart Hospital, 1 Singil-ro, Seoul 07441, Republic of Korea

**Keywords:** ceramide, chronic pruritus, gastrin-releasing peptide receptor (GRPR), neuroimmune crosstalk, sphingosine-1-phosphate, transient receptor potential ankyrin 1 (TRPA1)

## Abstract

Chronic pruritus is a complex sensory disorder arising from interactions among epidermal barrier dysfunction, immune signaling, peripheral sensory neurons, spinal circuits, and supraspinal networks. Bioactive lipids participate in this neuroimmune crosstalk as both structural components of the skin barrier and signaling mediators. Altered ceramide composition and sphingolipid metabolism accompany barrier dysfunction, while sphingosine-1-phosphate (S1P), ceramide-1-phosphate (C1P), eicosanoids, and autotaxin-derived lysophosphatidic acid (LPA) modulate inflammatory and sensory pathways. In particular, experimental studies implicate S1P–S1PR3–transient receptor potential (TRP) signaling and LPA receptor pathways in peripheral pruriception, although their causal relevance to human chronic itch remains incompletely established. These mechanisms interact with cytokines including interleukin (IL)-31, thymic stromal lymphopoietin, and IL-33 to enhance sensory-neuron responsiveness. Within the spinal dorsal horn, gastrin-releasing peptide (GRP)/GRPR-dependent circuits integrate pruriceptive input and are modulated by neuroimmune signaling, potentially contributing to central sensitization. Supraspinal sensory, affective, motivational, and reward-related networks may further reinforce the itch–scratch cycle. This review integrates lipid metabolism, neuroimmune signaling, and neural plasticity across peripheral, spinal, and supraspinal levels, with emphasis on atopic dermatitis (AD), prurigo nodularis (PN), psoriasis, and systemic pruritic disorders, and discusses therapeutic strategies and research priorities for mechanism-based treatment of chronic itch.

## 1. Introduction

Itch and pain are protective somatosensory experiences that share several peripheral and central signaling mechanisms but evoke distinct behavioral responses: withdrawal from painful stimuli and scratching in response to itch [1,2,3]. According to the International Forum for the Study of Itch (IFSI), chronic pruritus (CP) is defined as pruritus persisting for six weeks or longer [4]. Beyond this temporal definition, chronic itch differs from acute itch in that persistent pruriceptive input may be accompanied by sustained neuroimmune activation and functional changes in sensory processing, potentially contributing to symptom persistence [2,3,5]. CP can substantially impair sleep, psychological well-being, and quality of life [5,6]. A population-based study in Germany reported a point prevalence of 13.5% and a 12-month prevalence of 16.4% for chronic pruritus in adults [7]. However, the epidemiological burden varies according to population characteristics, age, and underlying disease. Chronic pruritus is particularly relevant in older adults and in patients with inflammatory dermatoses, including atopic dermatitis (AD) and psoriasis, as well as systemic disorders such as chronic kidney disease and cholestatic liver disease [5,8,9].

Historically, itch research focused largely on histamine-dependent mechanisms and cutaneous inflammation. Histaminergic itch is mediated primarily through histamine receptors on sensory neurons, whereas non-histaminergic itch involves diverse mediators, including cytokines, proteases, and bioactive lipids [10,11]. Accordingly, responses to antihistamines vary substantially across pruritic diseases. H1-antihistamines are generally effective for histamine-mediated conditions such as urticaria but often provide limited relief in chronic pruritic disorders such as AD, prurigo nodularis (PN), and chronic kidney disease-associated pruritus, in which non-histaminergic pathways play important roles [5,8,10]. These differences highlight the need to investigate alternative neuroimmune and lipid-mediated mechanisms underlying chronic itch [11].

Lipid biology is increasingly recognized as an important component of this network [12]. Epidermal ceramides are essential for permeability-barrier integrity, whereas bioactive sphingolipids, eicosanoids, and lysophospholipids can modify inflammatory responses and peripheral sensory-neuron excitability [12,13]. At the spinal level, the relationship with lipid dysregulation is less direct; persistent peripheral pruriceptive and inflammatory input may promote neuronal and glial sensitization and remodeling of itch circuits [11,14]. At the supraspinal level, current evidence primarily supports altered processing of persistent pruriceptive input rather than a direct effect of cutaneous lipid abnormalities on cortical itch networks [15,16].

Although previous reviews have extensively addressed the immunological and neuronal mechanisms of chronic itch, the role of bioactive lipids as potential modulators at the interface between epidermal barrier dysfunction, inflammation, and sensory signaling has received comparatively less integrated attention. We therefore adopted a skin-to-central nervous system framework to examine how lipid-associated peripheral mechanisms relate to downstream spinal and supraspinal processing, while recognizing that the strength and directness of evidence differ across these levels.

In this review, we organize current evidence into three interconnected levels: (1) peripheral neuroimmune–lipid signaling in the skin; (2) spinal processing and neuroimmune amplification within the dorsal horn; and (3) supraspinal processing and neural plasticity associated with itch chronification, with particular attention to distinguishing direct lipid-mediated mechanisms from downstream neural consequences and to identifying where evidence is predominantly preclinical or indirect. We then examine how these mechanisms converge in major pruritic diseases and discuss their implications for mechanism-based treatment.

## 2. Literature Search Strategy, Study Selection, and Evidence Appraisal

This narrative review was developed through a structured literature search using PubMed/MEDLINE, focusing on neuroimmune and lipid-mediated mechanisms of chronic itch across peripheral, spinal, and supraspinal levels. Relevant English-language publications were identified using combinations of terms related to chronic itch or pruritus; bioactive lipids and lipid mediators, including ceramides, sphingosine-1-phosphate (S1P), ceramide-1-phosphate (C1P), lysophosphatidic acid (LPA), eicosanoids, and platelet-activating factor (PAF); sensory receptors and neurons; neuroimmune signaling; spinal itch circuits; glial activation; and supraspinal or cortical processing. Disease-specific terms, including atopic dermatitis (AD), prurigo nodularis (PN), psoriasis, chronic kidney disease-associated pruritus, and cholestatic pruritus, were also used where relevant. Reference lists of relevant publications were additionally examined to identify pertinent studies.

Original experimental studies, including in vitro and animal investigations, human observational studies, clinical trials, and relevant reviews, consensus statements, and guidelines were considered when they directly informed the mechanistic or clinical framework of this review. Literature selection was guided by relevance to lipid-mediated peripheral signaling, downstream spinal neuroimmune mechanisms, supraspinal processing, and disease-specific or therapeutic implications of chronic itch. Priority was given to studies providing direct mechanistic evidence, disease-specific human findings, or clinically relevant therapeutic outcomes. Studies addressing pain, general neuroinflammation, or other related processes without itch-specific outcomes were considered only when they provided relevant mechanistic context, and their indirect applicability to chronic itch was explicitly acknowledged. Publications lacking a clear relationship to the objectives of this review were not prioritized.

Evidence was synthesized qualitatively according to study design, experimental context, mechanistic relevance, and clinical applicability. Findings were distinguished as originating from in vitro experiments, animal/preclinical models, human observational studies, or interventional/clinical investigations. Particular attention was given to differentiating experimentally demonstrated mechanisms from associations observed in human studies and to identifying whether proposed lipid-mediated pathways were directly implicated in pruriception or represented downstream neuroimmune processes. Evidence from experimental pain or neuroinflammatory models was not considered sufficient to establish an itch-specific mechanism. The major studies informing the neuroimmune–lipid framework, including their experimental models, principal findings, relevance to chronic itch, and limitations, are summarized in Supplementary Table S1.

Because this article is a narrative rather than a systematic review, a formal systematic risk-of-bias assessment or GRADE evaluation was not performed. The Scale for the Assessment of Narrative Review Articles (SANRA) provides a framework for evaluating the quality of narrative reviews, whereas the Joanna Briggs Institute (JBI) critical appraisal tools, Newcastle–Ottawa Scale (NOS), and Critical Appraisal Skills Programme (CASP) checklists are used for critical appraisal of individual studies according to their designs. These instruments were not formally applied in the present review. Accordingly, the evidence synthesis is interpretative rather than a systematic quality-weighted assessment, and potential selection bias and heterogeneity in the supporting literature are acknowledged as methodological limitations.

## 3. Tier 1—Peripheral Skin: Bioactive Lipids as Neuroimmune Mediators

### 3.1. Epidermal Ceramide Homeostasis and Barrier Dysfunction

**Figure 1:**
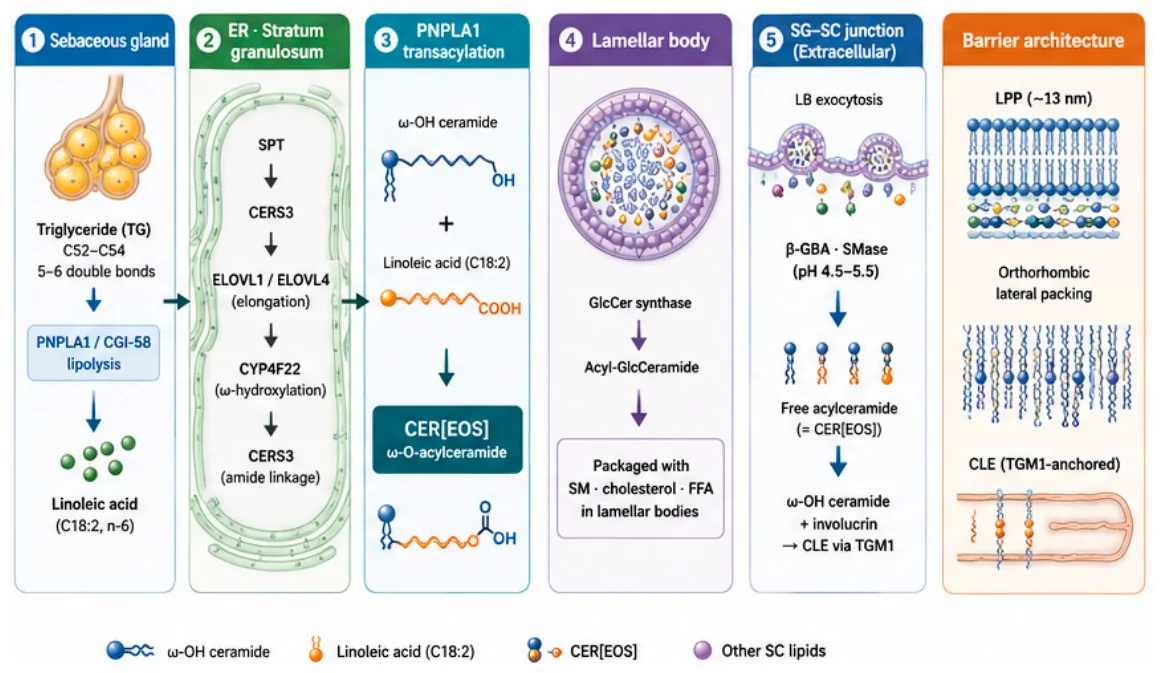
Biosynthetic pathway of epidermal acylceramide (ω-O-acylceramide). Schematic overview of the sequential pathway leading to the formation of epidermal ω-O-acylceramide and the stratum corneum lipid barrier. Linoleic acid derived from triglyceride reservoirs contributes to acylceramide synthesis in keratinocytes. During de novo ceramide synthesis in the endoplasmic reticulum, fatty acids are elongated by ELOVL enzymes, ω-hydroxylated by CYP4F22, and incorporated into ω-hydroxyceramides by CERS3. PNPLA1 subsequently esterifies linoleic acid to the ω-hydroxyl group, generating CER[EOS]. Acylceramides are glucosylated and packaged with other lipids into lamellar bodies, followed by secretion at the stratum granulosum–stratum corneum junction. Extracellular lipid processing generates the organized lamellar lipid matrix, whereas ω-hydroxyceramides become covalently attached to the cornified envelope to form the corneocyte lipid envelope (CLE). Together, CER[EOS]-dependent lamellar organization and CLE formation support the long periodicity phase (LPP), orthorhombic lipid packing, and epidermal permeability barrier. Arrows indicate the direction of lipid biosynthesis, intracellular transport, and extracellular processing. Different colors distinguish the major stages of acylceramide biosynthesis and barrier formation, while the colored lipid structures represent different lipid species, as indicated in the figure.

The stratum corneum (SC) lipid matrix consists predominantly of ceramides, free fatty acids, and cholesterol and is essential for maintaining epidermal permeability-barrier function. Epidermal ceramide synthesis involves coordinated fatty-acid elongation, ω-hydroxylation, ceramide synthesis, and acylation. Very-long-chain fatty acid elongase (ELOVL) enzymes generate long- and ultra-long-chain fatty acids, while cytochrome P450 family 4 subfamily F member 22 (CYP4F22) mediates ω-hydroxylation, ceramide synthase 3 (CERS3) incorporates these fatty acids into ceramide species, and patatin-like phospholipase domain-containing protein 1 (PNPLA1) catalyzes the formation of ω-O-acylceramides such as esterified omega-hydroxyacyl-sphingosine (CER[EOS]) [17,18]. The major pathways linking epidermal lipid metabolism, barrier dysfunction, and peripheral pruriceptive signaling are summarized in Figure 1.

**Figure 2:**
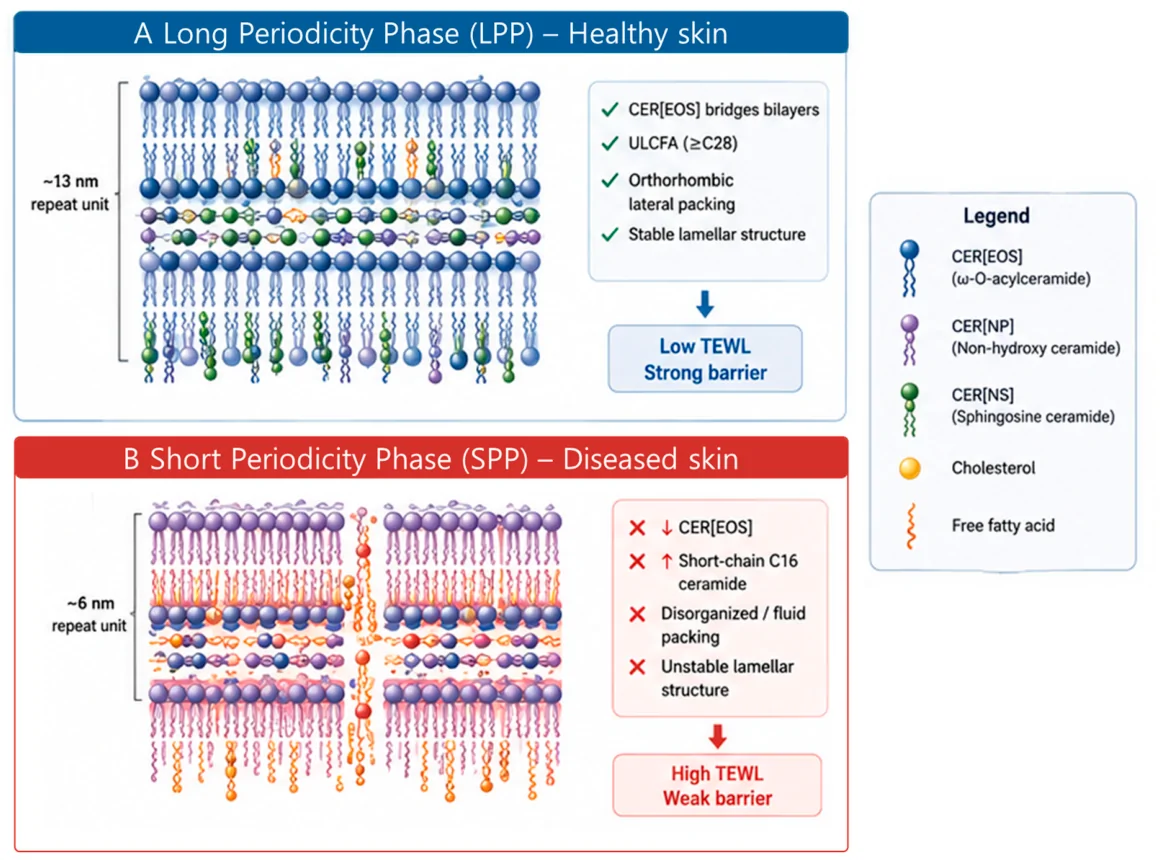
Stratum corneum lamellar lipid organization in healthy and diseased skin. Schematic comparison of the long periodicity phase (LPP) characteristic of an intact epidermal barrier and the short periodicity phase (SPP) favored by disease-associated lipid remodeling. (**A**) In healthy stratum corneum, CER[EOS] containing ultra-long-chain fatty acids contributes to bridging adjacent lipid layers and stabilizing the approximately 13 nm LPP. Together with cholesterol, free fatty acids, and other ceramide subclasses, this organization promotes dense orthorhombic lipid packing and low permeability. (**B**) Reduction of CER[EOS] and ultra-long-chain ceramides together with accumulation of shorter-chain ceramides disrupts interlamellar organization and favors an approximately 6 nm SPP with less ordered lipid packing. This transition weakens the permeability barrier, resulting in increased TEWL and enhanced penetration of environmental irritants and allergens. Red shading highlights the disrupted lipid organization and impaired barrier function in diseased skin. Arrows indicate the resulting changes in transepidermal water loss (TEWL), while red cross marks (×) denote alterations associated with impaired lipid organization and barrier stability.

Evidence from human AD skin and experimental models indicates that type 2 cytokines, particularly interleukin [IL]-4 and IL-13, alter epidermal lipid metabolism through STAT6-dependent suppression of fatty acid elongation pathways, thereby reducing long- and very-long-chain ceramide production [19,20,21]. In experimental mouse models, IFN-γ has also been shown to modify epidermal fatty-acid and ceramide metabolism [22]. Thymic stromal lymphopoietin (TSLP) has additionally been implicated in the suppression of arachidonate lipoxygenase 3 (ALOXE3) and ALOX12B, two epidermal lipoxygenases required for acylceramide processing and the subsequent formation of protein-bound ceramides [23]. Together with cytokine-mediated alterations in fatty-acid elongation and ceramide synthesis, these changes are associated with depletion of barrier-relevant ceramide subclasses, including non-hydroxy fatty acid–phytosphingosine ceramide (CER[NP]) and CER[EOS], and a shift toward shorter-chain ceramide species [17,24]. Such compositional changes have important consequences for SC lipid organization. In healthy SC, ultra-long-chain acylceramides, particularly CER[EOS], are critical for formation of the approximately 13 nm long periodicity phase (LPP), whereas ceramide abnormalities associated with AD can disturb lamellar organization and favor less ordered lipid packing, thereby increasing permeability to water and environmental substances [17,25]. The relationship between inflammatory cytokine signaling, altered ceramide metabolism, and disruption of SC lipid organization is illustrated in Figure 2.

Preclinical evidence summarized in previous studies indicates that increased SC pH and associated protease activity can impair lipid-processing enzymes, including β-glucocerebrosidase (β-GBA) and acidic sphingomyelinase (SMase), thereby reducing ceramide generation and further compromising barrier function [26]. These enzymes normally generate ceramides from glucosylceramide and sphingomyelin precursors delivered by lamellar bodies. Their reduced activity can therefore further limit ceramide availability, perpetuating barrier dysfunction through a self-amplifying cycle [26]. In parallel, transglutaminase 1 (TGM1) covalently attaches ω-hydroxyceramides to cornified-envelope proteins, including involucrin and envoplakin, to form the corneocyte lipid envelope (CLE), which provides a scaffold for the organization and anchoring of extracellular lamellar membranes [17]. Consequently, impaired TGM1-dependent lipid attachment or defective SMase-mediated ceramide generation can compromise CLE and barrier integrity through mechanisms that are distinct from alterations in ceramide chain length [17].

Beyond these structural consequences, altered ceramide homeostasis may also influence bioactive sphingolipid signaling. Ceramide deficiency may modify sphingolipid metabolic flux and favor increased sphingosine-1-phosphate (S1P) signaling through changes in sphingosine metabolism and sphingosine kinase activity. However, a direct causal sequence linking epidermal ceramide depletion to increased epidermal or dermal S1P has not been consistently demonstrated in human atopic skin [13]. In a parallel metabolic pathway, ceramide kinase (CERK) phosphorylates ceramide to generate ceramide-1-phosphate (C1P), which promotes inflammatory lipid signaling through activation and membrane recruitment of cytosolic phospholipase A2α (cPLA2α). S1P and C1P therefore represent bioactive sphingolipid mediators through which altered sphingolipid metabolism may extend beyond structural barrier dysfunction to inflammatory and pruriceptive signaling, as discussed in the following sections.

### 3.2. The S1P–S1PR3–TRPA1 Pruriceptive Axis

#### 3.2.1. Receptor Pharmacology and In Vitro Evidence

S1P signals through five G-protein-coupled receptors, S1PR1–5, which exhibit distinct expression patterns across cell types. In small-diameter dorsal root ganglion (DRG) neurons, a sensory-neuronal population enriched in pruriceptors and nociceptors, S1PR1 and S1PR3 are among the predominant S1P receptor subtypes, whereas S1PR2 is more prominently expressed in larger myelinated neurons [27]. These receptor-expression patterns have been characterized predominantly in experimental rodent sensory neurons, whereas directly comparable expression data in human DRG pruriceptor populations remain limited. In vitro patch-clamp and calcium imaging studies using cultured mouse DRG neurons have shown that S1P potentiates capsaicin-evoked currents through S1PR1-dependent phosphoinositide 3-kinase (PI3K)–p38 signaling, thereby enhancing TRPV1 responsiveness [28].

Importantly, S1PR3 mediates a distinct concentration-dependent response. In cultured mouse sensory neurons, nanomolar concentrations of S1P activate S1PR3 signaling through Gβγ, phospholipase C, and protein kinase Cε, leading to transient receptor potential ankyrin 1 (TRPA1) sensitization, Ca^2+^ influx, and action-potential generation in pruriceptive neurons [29]. Consistent with these cellular findings, in vivo mouse experiments demonstrated concentration-dependent S1P-evoked itch and pain behaviors, with TRPA1 selectively required for S1P-evoked itch and TRPV1 for acute pain and heat hypersensitivity [29]. Together these findings support a concentration-dependent role of S1PR3 signaling in itch- and pain-related responses in mice, although its relevance to human itch–pain discrimination remains to be established.

#### 3.2.2. In Vivo Evidence and Clinical Correlation

Consistent with these cellular findings, intradermal administration of low-dose S1P induces scratching behavior in conscious mice with minimal nociceptive responses, whereas higher doses evoke both scratching and pain-related behaviors in an S1PR3-dependent manner [29]. In these mouse models, genetic deletion or pharmacological inhibition of S1PR3 markedly reduces S1P-evoked scratching, supporting a functional role for the S1PR3–TRPA1 pathway in peripheral pruriception [29].

Clinical observations provide additional, although less direct, support for the relevance of altered sphingolipid signaling to inflammatory itch. Serum S1P levels have been reported to be elevated in patients with AD and to correlate with disease severity [30,31], while reduced ceramide abundance and altered ceramide composition are well-established features of AD skin [32]. Importantly, elevated circulating S1P does not necessarily reflect increased local S1P concentrations or signaling within the skin. Systemic S1P alterations and local cutaneous sphingolipid dysregulation should therefore be considered distinct, although potentially related, components of AD pathophysiology. However, a direct metabolic relationship linking cutaneous ceramide depletion to increased S1P signaling and clinical itch severity has not yet been established in human disease [33].

Collectively, these findings suggest a potential link between altered sphingolipid metabolism and peripheral pruriceptor activation, although a direct causal relationship has not been established in human chronic itch.

#### 3.2.3. Potential Spinal Relevance of S1P Signaling

S1P signaling is also present within the spinal cord, where sphingosine kinase 1/2 (SphK1/2) and S1P receptors are expressed in glial populations, including astrocytes. However, the direct contribution of spinal S1P signaling to chronic itch is less well established than its peripheral sensory effects.

Activation of astrocytic S1PR1 has been shown to promote NLRP3 inflammasome signaling, IL-1β and tumor necrosis factor (TNF)-α release, disruption of glutamatergic homeostasis, and increased dorsal horn neuronal excitability in experimental neuroinflammatory and neuropathic pain settings [34]. These findings demonstrate a potential mechanism by which spinal S1P signaling can modulate neuronal excitability, but they do not establish an itch-specific S1P pathway. Conversely, pharmacological blockade of spinal S1PR1 reduces astrocytic neuroinflammation and promotes IL-10 release in preclinical models [30]. Whether these spinal effects translate into therapeutic benefit for chronic itch remains to be determined [34].

### 3.3. Ceramide-1-Phosphate, cPLA2α, and Eicosanoid-Mediated Sensitization

C1P, generated through phosphorylation of ceramide by CERK, directly interacts with cPLA2α and promotes its translocation to cellular membranes, thereby facilitating arachidonic acid (AA) release [35]. Once released, AA serves as a substrate for multiple eicosanoid-generating pathways. Through the cyclooxygenase pathway, AA is converted to prostanoids, including prostaglandin E2 (PGE2) and prostacyclin (PGI2). These mediators can enhance the responsiveness of transient receptor potential vanilloid 1 (TRPV1)-expressing sensory neurons through EP1- and IP-receptor-dependent signaling involving protein kinase pathways, thereby lowering the threshold for TRPV1 activation [36]. However, these findings primarily demonstrate nociceptive neuronal sensitization rather than itch-specific pruriception. Direct evidence linking C1P-dependent prostanoid signaling to pruritic behavior or clinical itch remains limited.

AA can also be metabolized through the 5-lipoxygenase pathway to generate leukotrienes, including leukotriene B4 (LTB4). LTB4 acts through leukotriene B4 receptor 1 (BLT1) and BLT2 receptors and has been implicated in both sensory-neuron activation and recruitment of inflammatory cells, including eosinophils [12]. Thus, C1P-dependent mobilization of AA provides a potential biochemical link between sphingolipid metabolism, eicosanoid production, inflammation, and peripheral sensory sensitization.

In mouse models, LTB4 has also been directly implicated in itch-related behavior; pharmacological inhibition of 5-lipoxygenase or BLT receptors attenuated IL-31-induced scratching, while IL-31 increased LTB4 production in keratinocytes [37].

Importantly, this lipid–sensory interaction is not restricted to primary afferent neurons. Human keratinocytes express functional TRPV1 and TRPA1 channels and generate distinct eicosanoid profiles following their activation [38]. TRPA1 stimulation preferentially increases LTB4 production, whereas TRPV1 activation promotes PGE2 generation [38]. These findings suggest that TRP-channel activation in keratinocytes may modify the local eicosanoid environment and potentially influence inflammatory and sensory signaling. However, the proposed lipid-mediated amplification circuit remains largely inferred from experimental keratinocyte studies and has not been directly established as an integrated itch-specific pathway in vivo or in human pruritic skin.

From a therapeutic perspective, pharmacological inhibition of CERK has been shown to reduce C1P-dependent AA mobilization and downstream inflammatory responses in preclinical experimental systems [35,39,40].

### 3.4. Lysophosphatidic Acid and the Autotaxin Axis

#### 3.4.1. The ATX–LPA Axis in Cholestatic and Uremic Pruritus

LPA is a bioactive glycerophospholipid generated predominantly from lysophosphatidylcholine (LPC) by the secreted lysophospholipase D autotaxin (ATX; ENPP2), a major source of circulating LPA. Because ATX is a secreted enzyme, extracellular LPA production cannot necessarily be attributed to the cells expressing ATX. The relative contributions of keratinocytes, dermal fibroblasts, and infiltrating immune cells to local cutaneous LPA production remain incompletely defined, and a predominant cellular source has not been established in human pruritic skin. The ATX–LPA pathway has been particularly well characterized in cholestatic pruritus. In a landmark study using primary hepatocyte-conditioned media and sera from patients with primary biliary cholangitis (PBC) and intrahepatic cholestasis of pregnancy, LPA was identified as a potent Ca^2+^-mobilizing component of pruritic sera [41]. Serum ATX activity correlated strongly with itch intensity (r = 0.67, *p* < 0.0001) and showed a stronger association with pruritus than several candidate pruritogens, including bile salts, histamine, substance P, and endogenous opioids [41].

In contrast to cholestatic pruritus, for which the ATX–LPA axis has been relatively well characterized, the pathophysiology of uremic pruritus is multifactorial and remains incompletely understood [42]. Proposed mechanisms include systemic inflammation, neuropathic alterations, xerosis and skin-barrier dysfunction, and dysregulation of the endogenous opioid system [42]. Although renal dysfunction may also alter systemic lipid metabolism, direct evidence linking LPA accumulation or ATX–LPA signaling specifically to chronic kidney disease-associated pruritus (CKD-aP) remains limited. Thus, the role of the ATX–LPA pathway in CKD-aP should currently be regarded as less established than that in cholestatic pruritus.

#### 3.4.2. LPA Receptor Subtypes and Central Involvement

LPA exerts its biological effects through at least six G-protein-coupled receptors, LPAR1–6. In experimental models, intradermal LPA induces scratching behavior through LPAR5-dependent signaling, involving phospholipase D and downstream activation of TRPA1 and TRPV1 [43]. These experimental findings provide direct evidence that exogenously administered LPA can induce itch-related behavior in mice. However, they do not establish that locally produced cutaneous LPA drives spontaneous chronic pruritus in humans. LPA receptor subtypes may, however, exert divergent effects on pruriceptive signaling. In hydroxychloroquine-induced itch, Lpar5-deficient mice exhibit reduced scratching, whereas Lpar2-deficient mice show enhanced scratching, suggesting that the balance among LPA receptor subtypes can influence the magnitude of pruritic responses [44].

Notably, hydroxychloroquine-induced itch has also been associated with increased LPA levels in both the skin and thalamus [44]. These findings raise the possibility that LPA signaling is not restricted to peripheral pruriceptor activation but may also participate in central processing of itch. However, simultaneous changes in peripheral and central LPA concentrations do not establish a bidirectional LPA gradient or direct transport of LPA between the skin and brain. The relative contributions of locally generated versus circulating LPA to central itch pathways therefore remain to be determined.

LPA signaling may also occur locally within the skin. Cutaneous cells, including keratinocytes and fibroblasts, participate in local bioactive-lipid metabolism and signaling [12]. Such local lipid signaling may contribute to an LPA-rich microenvironment in inflamed or mechanically stressed skin, providing an additional mechanism by which lipid mediators could amplify peripheral sensory and inflammatory responses independently of changes in circulating LPA.

### 3.5. IL-31 and the Lipid Raft–Neuroimmune Interface

IL-31 is a member of the IL-6 cytokine family produced predominantly by activated Th2 cells, although additional cellular sources, including mast cells, eosinophils, and basophils, have also been described [45]. IL-31 signals through a heterodimeric receptor complex composed of IL-31RA and oncostatin M receptor β (OSMRβ), which is expressed by subsets of cutaneous sensory neurons [46]. Activation of neuronal IL-31RA/OSMRβ engages Janus kinase (JAK)-dependent signaling and downstream pathways that enhance the responsiveness of TRPV1- and TRPA1-expressing sensory neurons, thereby providing a direct molecular link between type 2 inflammation and peripheral pruriception [23,46,47].

IL-31 signaling also intersects with epidermal lipid homeostasis. In keratinocytes, IL-31 has been reported to suppress ELOVL3 expression, potentially limiting the synthesis of very-long-chain fatty acids required for normal epidermal ceramide composition [23]. Thus, IL-31 may simultaneously promote neuronal sensitization and contribute to epidermal lipid abnormalities. However, whether these effects form a self-sustaining feedback loop in human disease remains to be established.

Mast-cell-derived lipid mediators provide an additional interface between immune activation and sensory signaling. Platelet-activating factor (PAF), generated through phospholipase A2-dependent remodeling of membrane glycerophospholipids following FcεRI activation, can promote inflammatory cytokine production and signal through PAF receptors expressed by sensory neurons [12]. Although PAF may contribute to communication among mast cells, inflammatory cells, and peripheral sensory nerves, direct experimental evidence demonstrating that PAF mediates IL-31-induced pruriception is currently lacking. Therefore, PAF-associated inflammatory signaling and IL-31-dependent itch should be considered potentially interacting but mechanistically distinct pathways.

Other epithelial alarmins provide parallel routes of direct neuronal activation. IL-33, released following cellular stress or tissue injury, activates ST2-expressing peptidergic sensory neurons and induces rapid Ca^2+^-dependent itch responses [48]. TSLP, released from keratinocytes following barrier disruption and inflammatory stimulation, directly activates TRPA1-positive sensory neurons and induces itch through a TRPA1-dependent pathway [49]. Together with IL-31, these cytokines establish multiple routes through which epidermal and immune signals can directly increase peripheral sensory-neuron excitability.

TSLP may additionally link inflammatory signaling to epidermal lipid metabolism. TSLP has been reported to suppress ALOXE3 and ALOX12B, epidermal lipoxygenases involved in acylceramide processing and the subsequent formation of protein-bound ceramides within the corneocyte lipid envelope [23,24]. These findings suggest a potential barrier-neural mechanism through which TSLP may influence both epidermal lipid organization and peripheral sensory-neuron activation. However, direct human evidence demonstrating that TSLP-induced alterations in epidermal lipid metabolism contribute causally to itch remains lacking.

Beyond classical IgE-dependent mast-cell activation, Mas-related G protein-coupled receptor X2 (MRGPRX2) provides another mechanism for bidirectional communication between sensory nerves and mast cells. Substance P and other neuropeptides released from activated sensory fibers can stimulate MRGPRX2 on dermal mast cells, promoting mast-cell activation and the release of tryptase and other inflammatory mediators [50,51].

The clinical relevance of IL-31 signaling is particularly evident in PN. IL-31 expression is increased in PN lesions, and tissue IL-31 levels have been associated with itch severity [52]. In addition to IL-31 signaling, mast-cell activation and MRGPRX2-associated pathways have been implicated in the pathogenesis of chronic prurigo, further supporting bidirectional communication between cutaneous immune cells and sensory nerves in PN [53]. In the phase III OLYMPIA program, treatment with the humanized anti-IL-31RA monoclonal antibody nemolizumab produced substantially greater itch improvement than placebo, with separation between treatment groups occurring early after treatment initiation [52]. Histological and molecular analyses of nemolizumab-treated PN skin further demonstrated modulation of neuroimmune and epithelial markers, including IL-31-, TSLP-, and calcitonin gene-related peptide (CGRP)-associated changes and alterations in cutaneous nerve architecture [54]. These findings provide clinical support for IL-31RA signaling as a major neuroimmune pathway in PN while also illustrating that targeted inhibition of a pruritogenic cytokine pathway can influence multiple components of the cutaneous neuroimmune environment.

Beyond these cytokine-driven pathways, TRP-channel activity can also be modulated by the surrounding membrane lipid environment [55,56], while specialized pro-resolving lipid mediators such as resolvin D1 and D2 have been shown to inhibit TRPV1 and TRPA1 signaling through lipid-raft-dependent mechanisms [57]. Together, these preclinical and mechanistic findings suggest that membrane lipids and specialized pro-resolving mediators can modulate peripheral sensory-neuron activity. However, their therapeutic efficacy in patients with chronic pruritus has not been established.

The major bioactive lipid mediators linking epidermal dysfunction to peripheral pruriceptive signaling are summarized in Table 1.

## 4. Tier 2—Spinal Cord: Downstream Itch Circuits and Neuroimmune Amplification

Compared with peripheral mechanisms, direct evidence linking specific lipid abnormalities to spinal itch circuits remains limited. The pathways discussed in this section therefore represent downstream neural and neuroimmune mechanisms that may be influenced by persistent peripheral pruriceptive and inflammatory input, rather than established lipid-specific spinal pathways. Where relevant, potential links with bioactive lipid signaling are discussed separately.

### 4.1. The Spinal Itch-Relay Circuit

The spinal dorsal horn (SDH) processes pruriceptive signals through specialized interneuronal circuits involving gastrin-releasing peptide (GRP) and its receptor GRPR [58,59]. Human itch is mediated by distinct populations of primary sensory afferents. Histamine-induced itch is primarily transmitted by mechano-insensitive C-fibers [60], whereas non-histaminergic itch, such as cowhage-induced pruritus, involves mechano-responsive polymodal C-fibers [61]. These findings demonstrate that human pruriception involves multiple peripheral sensory pathways rather than a single histaminergic afferent population. These primary afferents transmit pruriceptive information to the SDH through neuropeptide- and glutamate-dependent pathways. In mice, natriuretic polypeptide B (NPPB), encoded by *Nppb*, functions as a key primary-afferent pruritogenic transmitter and acts upstream of GRPR-dependent spinal circuits [62]. In murine models, GRP activates GRPR-expressing interneurons within the dorsal horn, forming a key spinal relay for itch transmission [58,59,63]. However, the functional organization and relative contributions of NPPB and GRP/GRPR signaling to human spinal itch processing remain incompletely characterized.

GRPR-expressing neurons are concentrated in the superficial dorsal horn and comprise morphologically and functionally distinct interneuronal populations involved in the integration of pruriceptive input [64]. Excitatory glutamatergic transmission further contributes to activation of these circuits. Electrophysiological studies in experimental models have shown that GRP-dependent excitation of GRPR-expressing neurons is activity-dependent, with repetitive or high-frequency input facilitating recruitment of the GRP–GRPR pathway [63]. This activity dependence provides a potential mechanism by which sustained peripheral pruriceptor activity could strengthen spinal itch transmission during chronic itch, although the extent to which specific C-fiber firing patterns drive this process in human disease remains unclear.

In experimental chronic itch models, including acetone–ether–water-induced dry skin, DNFB-induced contact dermatitis, and MC903-induced AD-like dermatitis, excitatory synaptic transmission onto spinal GRPR-expressing neurons is enhanced. Neuronal pentraxin 2 (NPTX2), an activity-dependent synaptic protein, contributes to this plasticity by promoting clustering of AMPA-type glutamate receptors, including GluA1, at excitatory synapses onto GRPR-expressing neurons [14]. Genetic loss of *NPTX2* reduces this synaptic facilitation and attenuates chronic scratching behavior, supporting NPTX2-dependent excitatory synaptic remodeling as a mechanism contributing to spinal sensitization in chronic itch [14]. Although these findings support a role for NPTX2-dependent synaptic plasticity in itch-related spinal circuits, it remains unclear whether this mechanism is specific to pruriception or reflects broader activity-dependent neuronal sensitization.

Additional spinal neuronal populations modulate this GRPR-centered circuit. Neurokinin-1 receptor (NK1R)-expressing dorsal-horn neurons contribute to chronic itch transmission, and pharmacological disruption of NK1R signaling attenuates scratching in experimental chronic itch models [65]. Neuromedin B receptor (NMBR)- and GRPR-dependent pathways also interact within the dorsal horn. Cross-inhibition between NMBR- and GRPR-expressing circuits contributes to the regulation of histaminergic itch transmission, indicating that spinal pruriceptive output is determined by interactions among multiple excitatory and inhibitory interneuronal pathways rather than by a single linear GRP–GRPR relay [66].

Importantly, GRP/GRPR signaling is a well-established component of spinal itch circuitry in experimental models, but direct evidence linking cutaneous lipid dysregulation to activation or remodeling of this pathway remains limited. In the present framework, GRP/GRPR signaling is therefore considered primarily as a downstream spinal integrator of sustained peripheral pruriceptive input.

### 4.2. Microglial NLRP3–IL-1β–GRPR Amplification

A 2023 study by Liu et al. provided direct evidence that spinal microglia contribute to amplification of the itch-relay circuit in experimental chronic itch [67]. Across multiple mouse models, including house dust mite-induced AD-like dermatitis, DNFB-induced allergic contact dermatitis, acetone–ether–water-induced dry skin, and imiquimod-induced itch, activation of the NLRP3 inflammasome and increased IL-1β signaling were observed in spinal microglia [67]. Pharmacological inhibition of the NLRP3–caspase-1 pathway with MCC950 or YVAD reduced scratching behavior and attenuated activation of GRPR-expressing spinal neurons. Conversely, intrathecal administration of recombinant IL-1β restored scratching responses following NLRP3 inhibition, supporting a functional role for microglial IL-1β in chronic itch amplification [67].

Mechanistically, microglia-derived IL-1β acts through IL-1R1 on GRPR-expressing dorsal-horn neurons and enhances their excitatory synaptic and neuronal responses [67]. These findings identify the NLRP3–IL-1β–GRPR pathway as a preclinical microglial-neuronal mechanism capable of increasing the gain of spinal pruriceptive transmission during chronic itch. However, a direct mechanistic link between cutaneous lipid dysregulation and activation of this spinal pathway has not been established.

TLR4-dependent glial signaling provides an additional mechanism of spinal neuroimmune modulation. In experimental chronic itch, TLR4 deficiency reduces scratching and alloknesis and attenuates spinal glial activation, supporting a role for TLR4-dependent neuroinflammatory signaling in itch sensitization [68]. However, these findings primarily establish a role for TLR4 in spinal itch-related neuroimmune responses rather than a direct lipid-mediated mechanism. The endogenous ligands responsible for spinal TLR4 activation and whether peripheral barrier dysfunction or lipid dysregulation contributes to this pathway remain unclear.

### 4.3. Astrocyte Contributions and the Endocannabinoid Brake

Spinal astrocytes represent an additional glial mechanism contributing to the maintenance of chronic itch. In mouse models of atopic and contact dermatitis, persistent reactive astrogliosis develops in the SDH through activation of STAT3, and conditional disruption of astrocytic STAT3 or pharmacological inhibition of spinal STAT3 attenuates chronic scratching [69]. Lipocalin-2 (LCN2), a STAT3-dependent factor upregulated in reactive astrocytes, further enhances GRP-evoked scratching, identifying the astrocytic STAT3–LCN2 pathway as a mechanism capable of amplifying spinal pruriceptive transmission [69].

Subsequent work further demonstrated that persistent astrocytic STAT3 activation is driven by IL-6-dependent Ca^2+^ signaling involving inositol 1,4,5-trisphosphate receptor type 1 (IP3R1) and transient receptor potential canonical (TRPC) channels [70]. In a chronic itch mouse model, IL-6 expression was increased in DRG neurons, while DRG neuron-specific IL-6 knockdown, astrocyte-specific IP3R1 knockdown, and pharmacological inhibition of spinal TRPC signaling reduced LCN2 expression and chronic itch [70]. These findings provide a mechanistic link between sustained primary sensory-neuron activity and persistent astrocytic activation within the spinal itch circuit.

Endocannabinoid signaling represents a lipid-sensitive modulatory system that may further influence sensory-neuron excitability. The endocannabinoid 2-arachidonoylglycerol (2-AG) and related lipid mediators can modulate cannabinoid-receptor and TRP-channel signaling [71]. However, direct evidence establishing an astrocyte-derived 2-AG–CB1 pathway as a major endogenous brake on chronic spinal itch remains limited. The contribution of spinal endocannabinoid metabolism to itch chronification and its therapeutic potential therefore require further investigation.

These findings establish an astrocytic mechanism of chronic itch amplification in experimental models; however, a direct relationship between this STAT3–LCN2 pathway and peripheral or spinal lipid dysregulation has not been demonstrated.

### 4.4. Descending Modulation and Opioid Signaling

Ascending pruriceptive signals are subject to top-down modulation by descending neural pathways that influence dorsal-horn excitability. Serotonergic and opioidergic systems are important modulators of itch transmission, and their effects depend on receptor subtype and anatomical site [11]. In particular, activation of the μ-opioid receptor can promote or exacerbate itch, whereas activation of the κ-opioid receptor (KOR) generally suppresses pruriceptive signaling [11].

Opioid-receptor signaling is an important modulatory system in itch [11]. However, direct evidence that the peripheral bioactive lipids discussed above, including C1P, S1P, or LPA, regulate chronic itch specifically through opioid receptors remains limited. Thus, potential interactions between lipid biology and opioid signaling require direct experimental validation in itch-specific models.

Clinical evidence strongly supports KOR signaling as a therapeutic target in selected chronic pruritic disorders. Difelikefalin is a selective, peripherally restricted KOR agonist that significantly reduced itch intensity in a phase III randomized trial of patients with moderate-to-severe pruritus undergoing hemodialysis [72]. Nalfurafine, a centrally active KOR agonist, has also demonstrated antipruritic efficacy and is used clinically for refractory uremic pruritus in Japan [11]. Together, these findings demonstrate that KOR modulation can provide clinically meaningful suppression of chronic pruritus, although the anatomical sites and mechanisms of action differ among KOR agonists.

## 5. Tier 3—Supraspinal Processing: The Cortical Itch Network and Its Chronic Reorganization

In contrast to peripheral lipid-mediated mechanisms, current evidence does not establish a direct causal effect of cutaneous lipid abnormalities on supraspinal itch networks. The findings discussed in this section therefore primarily reflect central processing and plasticity associated with persistent pruriceptive input.

### 5.1. Ascending Pathways and the Supraspinal Itch Network

Pruriceptive signals processed within the SDH are transmitted to supraspinal structures through ascending pathways that include spinothalamic and spinoparabrachial projections. Spinothalamic pathways relay sensory-discriminative information through thalamic nuclei to somatosensory cortical regions, whereas spinoparabrachial projections engage the parabrachial nucleus and downstream limbic and autonomic structures, including the amygdala, hypothalamus, and brainstem, thereby contributing to the affective–motivational and autonomic dimensions of itch [11].

Functional neuroimaging studies further demonstrate that itch engages a distributed supraspinal network rather than a single cortical locus. A recent activation-likelihood estimation meta-analysis identified consistent itch-related activation in the thalamus, basal ganglia, insula, claustrum, and inferior frontal regions, together with deactivation in the paracentral lobule and medial frontal gyrus [73]. These findings support coordinated recruitment of sensory, salience, motivational, and motor-related networks during itch. Importantly, these regions are not considered itch-specific centers, as they also participate in broader sensory, affective, attentional, and motivational processing, including responses to pain and other salient stimuli. However, deactivation of frontal regions should not be interpreted as direct evidence of loss of inhibitory control over scratching, and its functional significance in chronic itch remains to be established.

### 5.2. Neuroimaging of Chronic Itch

Neuroimaging studies indicate that cerebral processing of experimentally evoked itch differs between patients with chronic inflammatory skin disease and healthy individuals. In patients with AD, histamine-evoked itch produces altered activation of thalamic, prefrontal, basal ganglia, insular, and sensorimotor regions compared with healthy controls [15,74]. These findings are consistent with disease-associated functional reorganization of central itch-processing networks, although they do not by themselves establish central sensitization as the cause of chronic itch.

Contextual cues and visually induced itch further recruit motivational and reward-associated networks. During contagious itch in patients with AD, increased activity has been demonstrated in the supplementary motor area, ventral striatum, and orbitofrontal cortex [75]. Activity related to itch intensity has also been observed in temporal and posterior opercular regions and the precuneus [75]. These observations suggest that cognitive, contextual, and reward-related processes can modulate itch perception and the urge to scratch, potentially contributing to persistence of the itch–scratch cycle.

### 5.3. Structural Neuroplasticity

Structural neuroimaging studies suggest that chronic inflammatory skin disease may also be accompanied by alterations in brain morphology. In a voxel-based morphometry study of patients with AD, reduced gray matter volume was identified in several regions, including the bilateral precentral gyri, right frontal pole, and right middle temporal gyrus [76]. Within the AD group, gray matter reductions in selected regions were associated with greater disease severity and increased circulating inflammatory markers, suggesting a relationship between chronic cutaneous inflammation and brain structural variation [76].

These findings should be interpreted cautiously because cross-sectional imaging cannot determine whether structural differences precede chronic itch, develop as a consequence of persistent symptoms and inflammation, or reflect broader disease-associated factors. Functional imaging studies nevertheless demonstrate altered activity and connectivity involving the ACC, insula, prefrontal cortex, and corticostriatal networks during itch in AD, suggesting that chronic itch is accompanied by both functional and structural changes across distributed brain networks [77].

Whether these abnormalities are reversible after effective antipruritic treatment remains unknown. Before these neuroimaging alterations can be assigned mechanistic or biomarker significance, their associations with clinical itch severity must be established and validated through longitudinal and interventional studies. In particular, neuroimaging assessments before and after effective antipruritic treatment are needed to determine whether changes in brain structure or functional connectivity track improvements in itch and reflect itch-related mechanisms rather than nonspecific consequences of chronic inflammation.

### 5.4. BNP/NPPB and Periostin as Peripheral–Neural Signaling Nodes

Natriuretic polypeptide B (NPPB/BNP) is an itch-associated neuropeptide expressed by subsets of primary sensory neurons. In mice, NPPB released from pruriceptive sensory neurons acts upstream of GRP/GRPR-dependent spinal signaling and is required for normal behavioral responses to multiple pruritogens [62]. More recently, increased circulating BNP concentrations have been reported across several chronic pruritic disorders, including AD and PN, suggesting that systemic BNP may also reflect broader chronic itch activity [78].

BNP additionally participates in peripheral skin–nerve communication. Experimental studies have shown that IL-31 increases NPPB expression and BNP release from sensory neurons, while BNP can act on skin cells to induce proinflammatory and itch-associated responses [79]. These preclinical findings suggest a potential bidirectional communication mechanism between sensory neurons and cutaneous cells through BNP signaling. However, the functional contribution of this pathway to human chronic pruritus remains unconfirmed, and a complete skin–spinal cord–skin feedback loop has not been demonstrated in humans.

Periostin provides a complementary link between tissue remodeling and sensory-neuron activation. Periostin directly activates integrin αVβ3 expressed by a subset of pruriceptive sensory neurons and induces itch across several mammalian species [80]. Keratinocyte periostin production is enhanced by TSLP-dependent signaling, supporting a reciprocal interaction between epithelial inflammation, extracellular-matrix remodeling, and sensory-neuron activation [16]. Thus, BNP and periostin illustrate distinct mechanisms through which peripheral inflammatory and structural signals can interact with neuronal itch pathways.

## 6. Disease-Specific Neuroimmune–Lipid Itch Profiles

### 6.1. Atopic Dermatitis

AD provides a prototypical example of the interaction among epidermal barrier dysfunction, type 2 inflammation, peripheral sensory-neuron sensitization, and central itch processing [81]. At the peripheral level, IL-4 and IL-13 alter epidermal lipid metabolism through STAT6-dependent pathways, including suppression of elongases involved in long- and very-long-chain fatty-acid synthesis, thereby contributing to depletion of barrier-relevant ceramide subclasses such as CER[NP] and CER[EOS] [19,20]. IFN-γ can further modify epidermal fatty-acid and ceramide metabolism during chronic inflammation [22].

Additional abnormalities in sphingolipid metabolism may further compromise the epidermal barrier. Sphingomyelin deacylase activity has been implicated in abnormal sphingomyelin metabolism in AD, potentially diverting sphingomyelin away from ceramide-generating pathways and thereby contributing to reduced epidermal ceramide availability [23]. Altered ceramide homeostasis is accompanied by changes in S1P-related signaling, while preclinical studies have shown that S1PR3 activation sensitizes TRPA1-expressing pruriceptive neurons [13,29]. However, a direct quantitative relationship between cutaneous ceramide depletion, increased S1P generation, and itch severity in human AD remains to be established.

Type 2 cytokines provide an additional direct route to neuronal sensitization. IL-31 acts through IL-31RA/OSMRβ expressed by subsets of sensory neurons and enhances TRPV1- and TRPA1-dependent pruriceptive signaling [23,47]. TSLP and IL-33 similarly activate cutaneous sensory pathways, thereby linking epidermal inflammation to peripheral itch transmission [48,49].

Cutaneous microbial dysbiosis further amplifies this peripheral neuroimmune environment. *Staphylococcus aureus* colonization is common in AD and is associated with greater disease severity [82]. *S. aureus*-derived toxins, proteases, and other virulence factors can disrupt keratinocyte barrier function and promote epithelial inflammatory responses, including TSLP- and IL-33-associated signaling [82]. These events may increase peripheral sensory input and potentially contribute to downstream spinal neuroimmune sensitization. However, evidence linking cutaneous inflammation or barrier dysfunction directly to spinal glial activation remains largely indirect and preclinical. The specific signaling mechanisms connecting peripheral skin abnormalities to spinal neuroimmune responses have not been established in human chronic pruritus.

Clinical responses to targeted therapies provide translational support for this integrated model. Dupilumab, which blocks IL-4Rα-dependent IL-4/IL-13 signaling, improves epidermal barrier function and partially normalizes abnormal SC lipid and ceramide profiles in AD [19,83]. Nemolizumab directly blocks IL-31RA signaling and produces rapid reductions in itch while also modifying neuroimmune and epithelial markers in treated skin [52,54]. Together, these therapeutic observations illustrate how barrier-directed, immune-directed, and neuron-directed mechanisms converge on the clinical expression of chronic itch in AD.

### 6.2. Prurigo Nodularis

PN shares several type 2 inflammatory and pruritogenic pathways with AD but is distinguished by prominent extracellular-matrix remodeling, neurotrophic signaling, and structural alterations within lesional skin. Transcriptomic comparisons of PN and AD lesions have demonstrated increased expression of neurotrophic and inflammatory mediators, including nerve growth factor (NGF); matrix metalloproteinases such as MMP1, MMP3, MMP10, and MMP13; oncostatin M; IL-1 family cytokines; and chemokines in PN [54,84]. Pathway analyses further identify enrichment of extracellular-matrix disassembly, neurotrophin–TRK signaling, and tissue-remodeling programs, while immunohistochemistry demonstrates increased dermal NGF expression in PN lesions [84]. These findings support a close interaction between tissue remodeling and neural dysregulation in PN.

Cutaneous neural architecture is also altered in PN. Reduced intraepidermal nerve-fiber density has been reported in lesional skin, together with abnormalities in dermal nerve morphology and neuropeptide-associated signaling [84]. Clinically, alloknesis and hyperknesis are frequently observed, indicating enhanced mechanical and sensory responsiveness [85]. However, these features should be interpreted as evidence of peripheral and central sensitization rather than as proof that central sensitization is uniformly more severe in PN than in AD.

IL-31 signaling represents one of the best clinically validated neuroimmune pathways in PN. Increased IL-31 expression has been documented in PN, and blockade of IL-31RA with nemolizumab produces substantial and rapid reductions in itch [52]. In the OLYMPIA-2 phase III trial, a ≥4-point reduction in peak-pruritus numerical rating scale was achieved by 56.3% of nemolizumab-treated patients compared with 20.9% receiving placebo at week 16 [52]. Tissue studies further demonstrate that nemolizumab modifies IL-31-, TSLP-, and CGRP-associated pathways and alters cutaneous nerve-related markers [54].

Periostin provides an additional link between extracellular-matrix remodeling and pruriceptive signaling. Periostin is increased in type 2 inflammatory environments and can directly activate integrin αVβ3-containing receptors on sensory neurons to induce itch [80]. Periostin also participates in immune–stromal signaling capable of promoting IL-31 production, providing a potential mechanism by which tissue remodeling may reinforce neuroimmune itch pathways [86]. These observations are particularly relevant to PN, in which fibrosis, fibroblast activation, and extracellular-matrix abnormalities are prominent.

Systemic BNP concentrations are also increased in patients with PN and several other chronic pruritic disorders [78]. This association raises the possibility that BNP may serve as a marker of chronic pruritic activity or participate in broader skin–neural communication. However, the available human data do not establish that elevated circulating BNP reflects a spinal-to-skin feedback mechanism specifically in PN.

### 6.3. Cholestatic and Uremic Pruritus

Cholestatic and uremic pruritus illustrate how systemic biochemical abnormalities can engage peripheral sensory pathways in the absence of primary inflammatory skin disease. In cholestasis, the ATX–LPA pathway represents one of the best-characterized lipid-associated pruritogenic mechanisms. ATX generates LPA from circulating LPC, and serum ATX activity correlates closely with itch intensity in patients with cholestatic disease [41]. Changes in ATX activity have also been reported to parallel changes in pruritus during therapeutic interventions, providing strong clinical support for involvement of the ATX–LPA axis in cholestatic itch [41,42].

A second, mechanistically distinct pathway involves Mas-related G protein-coupled receptor X4 (MRGPRX4). MRGPRX4 is expressed in a subset of human DRG neurons and can be activated by bile acids, resulting in intracellular Ca^2+^ mobilization and neuronal excitation [87]. Intradermal administration of bile acids or an MRGPRX4-selective agonist also induces itch in human subjects, supporting MRGPRX4 as a human-relevant bile acid-sensing pruriceptor [87]. Bilirubin has also been shown to modulate MRGPRX4 activity in experimental systems, suggesting that multiple cholestasis-associated metabolites may interact with this receptor [87]. However, the clinical relevance of bilirubin-MRGPRX4 signaling in human cholestatic pruritus remains to be established.

By contrast, the role of LPA in CKD-aP is much less clearly established. Experimental studies demonstrate that LPAR5 promotes itch and that altered LPA concentrations can occur in peripheral and central tissues in pharmacologically induced itch models [44]. However, these findings do not demonstrate that impaired renal clearance of LPA is a major driver of CKD-aP in humans. Current evidence instead supports a multifactorial pathophysiology involving systemic inflammation, peripheral neuropathy, xerosis, and dysregulation of endogenous opioid signaling [88,89].

Opioid pathways are particularly relevant to systemic pruritus. μ-Opioid receptor activation can facilitate itch, whereas KOR activation generally exerts antipruritic effects [11,89]. This therapeutic principle has been clinically validated in CKD-aP: difelikefalin, a selective, peripherally restricted KOR agonist, significantly reduces itch in patients undergoing hemodialysis [72].

Nalfurafine, another KOR agonist, is used clinically for refractory uremic pruritus in Japan. Together, these observations demonstrate that opioid modulation represents an established therapeutic strategy for systemic pruritus, although its mechanism is distinct from the ATX–LPA pathway.

### 6.4. Psoriatic Pruritus

Psoriatic pruritus arises within an inflammatory environment dominated by the IL-23/IL-17 and TNF axes, but its sensory phenotype also reflects interactions among keratinocytes, neurotrophic mediators, peripheral sensory fibers, and bioactive lipids. S1P signaling is altered in psoriatic inflammation, and experimental modulation of the S1P pathway can influence keratinocyte survival, inflammatory responses, and psoriasis-like dermatitis [90]. However, the extent to which S1P directly drives clinical itch through the S1PR3–TRPA1 pathway in human psoriasis remains to be established.

Neurotrophic signaling provides a more direct link to psoriatic itch. NGF and its receptor TrkA are increased in pruritic psoriatic skin and correlate with itch severity [91]. These findings support an NGF–TRPV1-associated neurogenic component of psoriatic pruritus, although changes in cutaneous nerve-fiber density vary among studies.

Psoriatic inflammation is also accompanied by substantial alterations in epidermal ceramide metabolism. Compared with healthy skin, psoriatic SC contains a higher proportion of shorter-chain ceramides and reduced long-chain ceramide species, changes that are associated with impaired barrier organization [25]. Decreased expression of ELOVL1, ELOVL4, and CERS3 may further contribute to impaired epidermal lipid organization [92]. These lipid abnormalities provide a mechanistic link between inflammation and barrier dysfunction without requiring a psoriasis-specific increase in ELOVL1.

Bioactive oxylipins constitute an additional lipid component of psoriatic inflammation. Lipidomic profiling of psoriatic lesions has demonstrated marked alterations in arachidonic-acid-derived mediators and a significant increase in the linoleic-acid-derived oxylipin 13-hydroxyoctadecadienoic acid (13-HODE) compared with healthy skin [93]. Although oxidized linoleic-acid metabolites can modulate TRP-channel activity, the direct contribution of individual HODE species to clinical psoriatic itch remains incompletely defined. Thus, these metabolites are best regarded as candidate links between inflammatory lipid remodeling and sensory signaling rather than established pruritogens in psoriasis.

**Figure 3:**
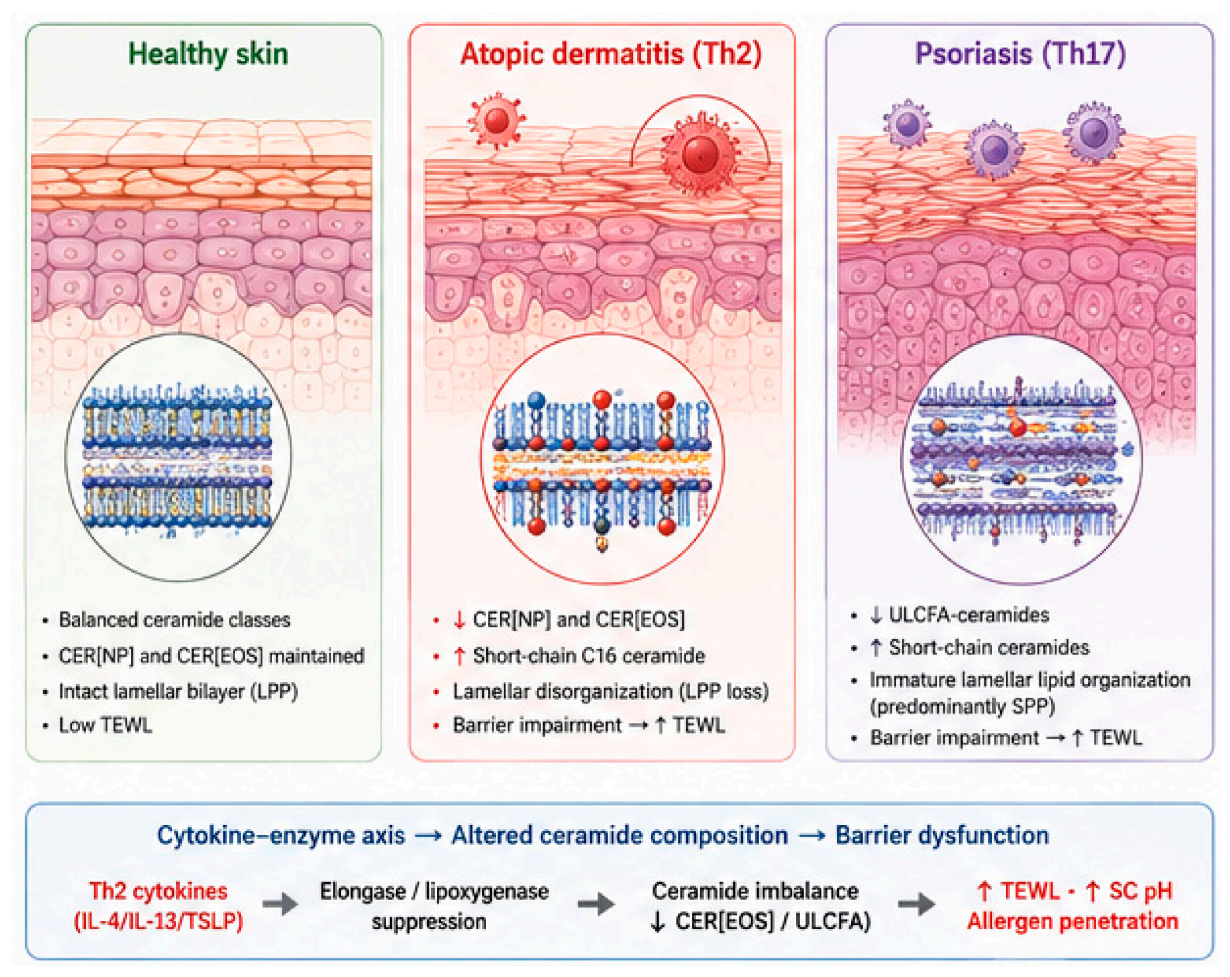
Disease-specific alterations in epidermal ceramide composition in inflammatory dermatoses. Schematic comparison of epidermal lipid organization in healthy skin, atopic dermatitis (AD), and psoriasis. Healthy skin maintains a balanced ceramide composition, including CER[NP] and CER[EOS], supporting an intact long periodicity phase (LPP) and low TEWL. In AD, Th2-dominant inflammation is associated with altered lipid-metabolic enzyme activity; depletion of long- and ultra-long-chain ceramides, including CER[EOS]; and relative enrichment of shorter-chain ceramides. These changes disrupt lamellar organization and increase TEWL. Psoriasis also exhibits altered ceramide composition and impaired lipid organization, although the inflammatory and metabolic context differs from that of AD. Despite disease-specific upstream mechanisms, altered ceramide composition converges on impaired lamellar organization, increased skin surface pH, enhanced permeability, and barrier dysfunction. Green, red, and purple indicate healthy skin, atopic dermatitis, and psoriasis, respectively. Gray arrows indicate the proposed progression from cytokine-mediated enzyme alterations to ceramide imbalance and barrier dysfunction, whereas red upward and downward arrows indicate increases and decreases in the corresponding parameters, respectively.

Despite disease-specific differences in upstream inflammation, psoriasis and AD share alterations in SC ceramide composition, including depletion of long- and ultra-long-chain ceramide species and disruption of barrier lipid organization [25]. These convergent alterations in epidermal lipid composition are illustrated in Figure 3. However, such lipid similarities may reflect shared epidermal barrier dysfunction rather than identical neuroimmune itch pathways. AD and psoriasis are characterized by distinct predominant inflammatory pathways, involving type 2 cytokines and IL-23/IL-17-associated signaling, respectively, which may differentially influence sensory-neuron activation. Therefore, similar ceramide abnormalities should not be interpreted as evidence of a common pruritogenic mechanism without direct disease-specific validation.

Importantly, epidermal lipid abnormalities in psoriasis are dynamic and responsive to cytokine-directed treatment. In the randomized SKINERGY-PP trial, 16 weeks of guselkumab treatment normalized the lesional SC ceramide profile toward that of healthy controls and improved barrier function, with changes in ceramide composition occurring in parallel with improvements in target-lesion severity and TEWL [94]. The ceramide profile and TEWL of non-lesional skin remained largely unchanged [94]. These observations provide clinical evidence that suppression of IL-23-driven inflammation can restore epidermal lipid homeostasis in psoriasis.

The PSORITUS phase IIIb randomized withdrawal study further demonstrated that secukinumab treatment markedly reduced psoriasis-associated pruritus and was accompanied by recovery of cutaneous nerve-fiber architecture [95]. Following treatment withdrawal, pruritus and disease activity increased in the placebo group, whereas the improvement in cutaneous neuroanatomy remained relatively stable, suggesting that IL-17A-directed therapy can modify both pruritic activity and peripheral neural alterations associated with psoriasis [95].

### 6.5. Comparative Disease Context of Lipid-Associated Pathways

The relative contribution of individual lipid pathways to chronic itch appears to vary across disease contexts, although direct head-to-head comparisons are lacking. S1P, C1P, and LPA should therefore not be interpreted as uniformly established drivers of itch across all pruritic disorders. Rather, the available evidence differs substantially with respect to disease context, experimental model, and degree of human validation. Accordingly, the current literature does not support a universal hierarchy of these pathways across chronic pruritic diseases; instead, the strength and nature of the evidence are pathway- and disease-dependent. Table 2 summarizes the disease-specific evidence for the major lipid-associated pathways discussed in this review and highlights areas in which mechanistic or clinical validation remains limited.

## 7. Therapeutic Implications

### 7.1. Tier 1: Peripheral Interventions

Restoration of epidermal barrier lipids represents the most direct strategy for targeting upstream abnormalities in the peripheral neuroimmune–lipid axis. Topical formulations containing ceramides, cholesterol, and free fatty acids can improve SC lipid organization and reduce TEWL, supporting their use as barrier-directed therapy in inflammatory skin disease [25]. However, direct evidence that topical ceramide replacement reduces epidermal S1P concentrations or sensory-nerve activation in patients with chronic itch remains limited.

Cytokine-directed therapies provide a complementary approach by suppressing inflammatory pathways that disrupt both epidermal lipid metabolism and neuronal signaling. Dupilumab, which blocks IL-4Rα-dependent IL-4/IL-13 signaling, improves epidermal barrier function and progressively normalizes abnormal SC lipid profiles in AD [83]. Pediatric data from the PELISTAD study further demonstrate recovery of lesional and non-lesional barrier function during dupilumab treatment [96]. Nemolizumab targets IL-31RA and thereby interrupts one of the most clinically validated cytokine–sensory-neuron pathways in AD and PN [52,54].

Other IL-13-directed biologics, including tralokinumab and lebrikizumab, effectively suppress type 2 inflammation and improve AD, but direct evidence that their clinical efficacy is mediated by restoration of specific ELOVL-dependent ceramide pathways or direct attenuation of neuronal sensitization remains limited. Their effects on epidermal lipid composition therefore warrant further mechanistic investigation.

The ATX–LPA pathway represents another potential peripheral therapeutic target, particularly in cholestatic pruritus, in which circulating ATX activity is strongly associated with itch intensity [41]. Although pharmacological ATX inhibition represents a mechanistically promising approach for LPA-associated itch, it should remain investigational until its antipruritic efficacy and safety are established in controlled clinical trials.

### 7.2. Tier 2: Spinal and Neural Interventions

Pharmacological modulation of neuronal signaling provides an important therapeutic strategy for chronic itch, although currently available agents act at different anatomical levels. The selective KOR agonist difelikefalin is peripherally restricted and has demonstrated clinically meaningful antipruritic efficacy in patients with CKD-aP undergoing hemodialysis [72]. Its clinical efficacy therefore supports peripheral KOR signaling as an established antipruritic target rather than providing direct evidence for pharmacological inhibition of spinal itch circuits.

JAK1-dependent signaling represents another potential link between inflammatory cytokines and sensory sensitization. In an AD-like mouse model, the JAK1-selective inhibitor abrocitinib and the JAK1/2 inhibitor baricitinib reduced mechanical alloknesis without detectable short-term improvement in dermatitis severity or skin-barrier function [97]. These findings suggest that JAK1 signaling contributes to mechanically evoked itch independently, at least in part, of its broader anti-inflammatory effects [97]. However, whether this effect reflects direct sensory-neuronal JAK1 inhibition, modulation of descending pathways, or additional neural mechanisms requires further investigation.

Gabapentinoids, including gabapentin and pregabalin, bind the α2δ subunit of voltage-gated calcium channels and reduce excitatory neurotransmitter release, providing a mechanistic basis for their use in neuropathic and centrally sensitized itch [98]. Their clinical utility is particularly relevant to neuropathic pruritic conditions, although direct inhibition of glutamatergic transmission specifically onto GRPR-expressing spinal neurons has not been established in humans.

Altered chloride homeostasis within the SDH represents an emerging mechanism of central sensory sensitization. KCC2 maintains low intracellular chloride concentrations required for effective GABAergic and glycinergic inhibition. In a DNFB-induced chronic itch model, pharmacological enhancement of Kcc2 expression with kenpaullone increased spinal KCC2 expression and markedly reduced scratching, suggesting that restoration of spinal inhibitory gating may represent a potential therapeutic strategy for chronic itch [99].

Microglial neuroimmune signaling provides another preclinical target. Inhibition of the NLRP3–caspase-1 pathway with MCC950 or YVAD reduces chronic scratching and suppresses IL-1β-dependent sensitization of GRPR-expressing spinal neurons in experimental chronic itch [67]. These findings identify the microglial NLRP3–IL-1β–GRPR pathway as a potential therapeutic target, although clinical validation in chronic pruritus is lacking.

Endocannabinoid signaling may provide an additional lipid-sensitive modulatory pathway, but direct evidence supporting spinal FAAH or MAGL inhibition as an antipruritic strategy remains insufficient. Further itch-specific studies are required before endocannabinoid augmentation can be prioritized as a therapeutic approach.

### 7.3. Tier 3: Supraspinal and Behavioral Interventions

The involvement of cognitive, affective, motivational, and reward-related brain networks in chronic itch provides a rationale for behavioral interventions targeting the itch–scratch cycle. Cognitive–behavioral approaches and habit-reversal training aim to modify attention to itch, habitual scratching responses, and behavioral reinforcement associated with scratching. Randomized studies and meta-analytic evidence suggest that behavioral interventions can reduce scratching and improve clinical outcomes in patients with AD [100]. However, direct evidence that these interventions restore prefrontal inhibitory activity or normalize frontostriatal circuitry in chronic itch remains limited.

Psychological and stress-related factors can also modulate itch perception, supporting the potential value of stress-reduction and mindfulness-based approaches as adjunctive strategies. Nevertheless, evidence that mindfulness specifically normalizes ACC activity in patients with chronic itch is currently insufficient, and neuroimaging studies directly evaluating treatment-related changes are needed.

Opioid modulation represents an additional pharmacological approach to selected chronic pruritic disorders. μ-Opioid receptor antagonists, including naltrexone, have demonstrated antipruritic activity in some clinical settings [89]. However, their therapeutic effects should not currently be attributed specifically to disruption of ACC–striatal scratch-reward circuitry, because this mechanism has not been directly established in patients with chronic itch.

Finally, neuroimaging has identified functional and structural alterations across sensory, salience, motivational, and cognitive-control networks in chronic itch [11,73]. At present, however, these findings remain primarily mechanistic and cannot yet be used to select individual patients for CNS-directed therapy. Longitudinal studies integrating neuroimaging with clinical treatment response will be required to determine whether brain-network characteristics can eventually serve as predictive or treatment-response biomarkers.

Importantly, the strength of clinical evidence for these interventions varies substantially across pruritic diseases. Clinical studies support cytokine-directed therapies in AD and PN, IL-23/IL-17-targeted therapies in psoriasis, and peripheral KOR agonism in CKD-associated pruritus, whereas the evidence for gabapentinoids is more condition-dependent [52,54,72,83,94,95]. These disease-specific findings should not be generalized to other forms of chronic itch without appropriate clinical validation.

Current and emerging therapeutic strategies targeting the neuroimmune–lipid itch axis are summarized in Table 3 and categorized according to the level of supporting evidence, clinical development status, and key translational limitations.

## 8. Knowledge Gaps and Research Priorities

Several mechanistic and translational questions remain unresolved. First, the relative contributions of S1P, C1P, and LPA to chronic itch across different disease contexts remain unclear. Comparative lipidomic studies in well-characterized patient cohorts, integrated with itch severity and sensory phenotypes, may help determine which lipid pathways are most relevant to individual pruritic disorders and most amenable to therapeutic intervention.

Second, although peripheral S1P signaling has been implicated in sensory-neuron activation and pruriception, the extent to which spinal or supraspinal sphingolipid signaling contributes independently to itch chronification remains poorly defined. Compartment- and cell-specific manipulation of sphingolipid signaling will be required to distinguish peripheral from central contributions and to determine whether changes in central lipid signaling arise independently or as a consequence of persistent peripheral input.

Third, the reversibility and clinical significance of chronic itch-associated brain alterations remain uncertain. Longitudinal neuroimaging studies before and after effective antipruritic treatment are needed to determine whether changes in the ACC, prefrontal cortex, and reward-related networks represent reversible neuroplastic adaptations and whether such changes can serve as biomarkers of treatment response.

Fourth, sex, aging, and hormonal status represent potentially important but insufficiently studied modifiers of the neuroimmune–lipid itch axis. Age- and hormone-associated changes in epidermal lipid homeostasis, barrier function, immune signaling, and sensory-neuron responsiveness may contribute to differences in chronic itch susceptibility and severity. Future studies should therefore incorporate sex and age as biological variables and directly examine their interactions with sphingolipid metabolism and pruriceptive signaling.

Fifth, several experimentally established pathways remain clinically underexplored. Peripheral mechanisms involving periostin–integrin αVβ3 and MRGPRX4 signaling, together with supraspinal reward-related circuits, represent potential targets for further investigation [16,80,87]. Although peripheral κ-opioid receptor agonism has demonstrated clinical antipruritic efficacy in hemodialysis-associated pruritus [72], the therapeutic relevance of other proposed pathways requires further validation. Future studies should clarify how peripheral, spinal, and supraspinal mechanisms interact to inform mechanism-based therapeutic approaches.

Sixth, the gut–skin–brain axis represents an emerging but incompletely characterized dimension of chronic itch. Microbial metabolites may influence epidermal barrier function, immune responses, and neural signaling; however, their specific roles in itch initiation, chronification, and resolution remain largely undefined. The demonstrated clinical efficacy of the aryl hydrocarbon receptor (AhR) agonist tapinarof in atopic dermatitis provides therapeutic evidence for the relevance of AhR signaling in inflammatory skin disease, supporting further investigation of microbiome-derived metabolites as potential modulators of the neuroimmune–lipid itch axis [101].

## 9. Future Directions

Future research should prioritize translating mechanistic insights into clinically validated approaches for chronic itch. Longitudinal and interventional studies integrating skin lipidomics, inflammatory mediators, epidermal barrier assessments, and sensory or neuroimaging measures with validated clinical itch endpoints are needed to clarify the temporal relationships among lipid dysregulation, neuroimmune signaling, and pruritus and to help establish causal mechanisms.

Biomarker-guided clinical trials should investigate whether patients with distinct barrier-associated, inflammatory, or neural sensitization-related phenotypes benefit from pathway-specific or combination therapies. Prospective studies should compare targeted monotherapies with rational combination strategies using predefined biomarker profiles and clinical outcomes, including itch severity, sleep disturbance, and quality of life. However, the clinical utility of these proposed biomarker-guided approaches remains to be established through prospective validation.

## 10. Strengths and Limitations of the Review

A major strength of this review is its integrative framework linking epidermal lipid metabolism, immune signaling, and peripheral-to-central neural mechanisms of chronic pruritus. By examining these interactions across AD, PN, psoriasis, and systemic pruritic disorders, this review provides a disease-oriented and translational perspective on established and emerging therapeutic pathways.

Several limitations should be acknowledged. First, although a structured literature search and qualitative evidence synthesis were undertaken, this narrative review did not employ a systematic review protocol, formal study-level critical appraisal using standardized assessment tools, or quantitative evidence grading. Therefore, potential selection bias and heterogeneity in the quality of the literature reviewed cannot be excluded. Second, many proposed mechanisms, particularly those involving lipid-dependent neuronal signaling and spinal neuroimmune interactions, are supported primarily by preclinical evidence, limiting their direct extrapolation to human chronic pruritus. Third, heterogeneity in disease phenotypes, lipidomic methodologies, and clinical outcome measures complicates comparisons across studies. Finally, most reported associations between lipid alterations and chronic itch have not been causally validated in humans.

## 11. Conclusions

Chronic pruritus arises from interconnected alterations in epidermal barrier and lipid homeostasis, immune signaling, peripheral sensory-neuron sensitization, spinal neuroimmune circuits, and supraspinal processing. Bioactive lipids, including S1P, C1P, LPA, and eicosanoids, may contribute to this network by linking epidermal and inflammatory changes to peripheral pruriceptive signaling, while persistent sensory input can promote spinal amplification and supraspinal plasticity. Importantly, current evidence most strongly supports direct lipid-mediated mechanisms at the peripheral level, whereas lipid-specific causal links to spinal and supraspinal changes remain comparatively limited and are largely supported by preclinical or indirect evidence.

The clinical efficacy of cytokine-directed therapies such as dupilumab and nemolizumab, restoration of epidermal lipid and neural abnormalities with targeted biologics in psoriasis, and antipruritic effects of peripheral KOR agonism illustrate the therapeutic relevance of targeting different components of this network. Longitudinal and interventional studies integrating mechanistic biomarkers, including lipidomic, inflammatory, and neurophysiological measures, with validated clinical itch endpoints are needed to determine whether changes in these pathways precede, accompany, or predict improvements in pruritus. Such studies will be essential to distinguish causal drivers from downstream consequences and to establish the clinical relevance of mechanism-based therapeutic strategies.

## Figures and Tables

**Table 1:** Key bioactive lipid mediators modulating peripheral neuroimmune and pruriceptive signaling.

Mediator	Principal Source/Formation	Receptor or Molecular Target	Peripheral Neuroimmune/Pruriceptive Mechanism	Potential Therapeutic Approach
S1P	Sphingosine phosphorylation by SphK1/2; keratinocytes and immune cells	S1PR3; S1PR1	Low-concentration S1PR3 signaling sensitizes TRPA1 through Gβγ–PLC–PKCε signaling; higher concentrations additionally recruit TRPV1-dependent responses	S1PR modulators; SphK inhibitors
C1P	Ceramide phosphorylation by CERK	cPLA2α (intracellular target)	Promotes cPLA2α membrane recruitment and AA release, facilitating downstream prostanoid and leukotriene production	CERK inhibitors (investigational)
LPA	ATX-mediated conversion of LPC; circulating and locally generated LPA	LPAR5 (itch-promoting); LPAR2 (modulatory/itch-inhibiting)	LPAR5–phospholipase D signaling engages TRPA1/TRPV1 in sensory neurons; altered LPA signaling has also been observed in central itch-related regions	ATX inhibitors (investigational); indirect modulation of the ATX–LPA axis
PGE2	AA metabolism through the COX pathway; keratinocytes and inflammatory cells	EP receptors	Enhances sensory-neuron responsiveness and can sensitize TRPV1 through EP-receptor-dependent protein kinase signaling	COX inhibitors; EP-receptor modulation (investigational)
LTB4	AA metabolism through the 5-LOX pathway; leukocytes and keratinocytes	BLT1/BLT2	Promotes inflammatory-cell recruitment and may enhance peripheral sensory signaling	5-LOX pathway or BLT-receptor inhibition (investigational)
PAF	Phospholipid remodeling through PLA2-dependent pathways; mast cells and other inflammatory cells	PAFR	Promotes inflammatory signaling and can activate sensory pathways; may contribute to cytokine-mediated neuroimmune communication	PAFR antagonists (investigational)
RvD1/D2	DHA-derived specialized pro-resolving mediators	ALX/FPR2 and GPR32-associated signaling; membrane lipid-raft modulation	Suppress TRPV1/TRPA1 activity, including through modification of membrane lipid-raft properties	Pro-resolving lipid mediator-based approaches (investigational)

**Table 2:** Comparative evidence for lipid-associated pathways across chronic pruritic diseases.

Lipid Pathway	Disease Context	Experimental Model/Main Finding	Human Evidence	Causal Status	Key Gap
Ceramide/barrier lipids	AD, psoriasis	Barrier lipid composition and organization are altered	Human studies demonstrate altered ceramide profiles; links to itch are mostly indirect [19,20,22,25,83,94]	Association; direct itch causality unestablished	Specific ceramide–itch relationships
S1P–S1PR3	AD, psoriasis; experimental itch	Mouse sensory neurons and behavioral models support S1PR3–TRP-dependent pruriception	Mainly associative evidence [28,29,30,32,33,90]	Preclinical causal evidence; no established human causality	Human mechanistic validation
C1P–cPLA2α	Inflammatory skin signaling	Preclinical studies support C1P-dependent arachidonic acid/eicosanoid signaling	Disease-specific human itch evidence limited [35,39,40]	Lipid-signaling mechanism supported; itch-specific causality unestablished	Direct human itch evidence
LPA–ATX	Cholestatic pruritus	Mouse studies support LPA-dependent pruriceptive mechanisms	Human ATX activity correlates with itch intensity [41,42,43,44]	Preclinical mechanistic support; human association	Generalizability beyond cholestasis
Eicosanoids/oxylipins	Psoriasis, inflammatory skin disease	Experimental studies report TRP-related sensory effects	Human lesional lipid alterations reported [12,36,38,93]	Pathway-specific preclinical evidence; human itch causality unclear	Itch-specific versus inflammatory effects
No dominant lipid pathway established	PN	Neuroimmune and neural alterations are more extensively characterized than individual lipid pathways	Direct disease-specific human lipid–itch evidence limited [52,54,84,85,86]	Lipid-specific causal pathway unestablished	PN-specific mechanistic studies

**Table 3:** Current and emerging therapeutic strategies targeting the neuroimmune–lipid axis.

Target/Intervention	Main Indication	Level of Evidence	Clinical Status	Key Limitation
IL-4Rα/Dupilumab	AD	Clinical–high	Approved	Lipid normalization is secondary to immune modulation; direct lipid-mediated antipruritic mechanism not established
IL-31RA/Nemolizumab	PN, AD	Clinical–high	Approved	Direct effect on lipid pathways remains unclear
IL-23p19/Guselkumab	Psoriasis	Clinical–high	Approved	Ceramide normalization accompanies disease improvement; causal contribution to itch reduction is uncertain
IL-17A/Secukinumab	Psoriasis	Clinical–high	Approved	Neural recovery accompanies clinical improvement, but direct neural mechanism is not established
KOR/Difelikefalin	CKD-aP	Clinical–high	Approved	Relevant to selected systemic pruritus; not a lipid-targeted therapy
KOR/Nalfurafine	Uremic pruritus	Clinical	Approved in Japan	Regional approval; central/peripheral mechanisms not fully resolved
α2δ/Gabapentinoids	Neuropathic/central itch	Clinical	Off-label	Evidence varies by pruritic condition; GRPR-specific effects unproven
ATX/ATX inhibitors	Cholestatic pruritus	Translational/early clinical	Investigational	ATX–LPA association is strong, but direct antipruritic efficacy of selective ATX inhibition remains unestablished
SC lipid barrier/Ceramide topical therapy	Inflammatory skin disease	Clinical/supportive	Clinical use	Direct effects on S1P or neuronal signaling remain uncertain
NLRP3/MCC950-like inhibitors	Chronic itch	Preclinical	Preclinical	Demonstrated mainly in animal models; no clinical validation
KCC2 enhancement	Chronic itch	Preclinical	Preclinical	Antipruritic efficacy demonstrated in experimental models only

## Data Availability

No new data were created or analyzed in this study. Data sharing is not applicable to this article.

## Supplementary Materials

The following supporting information can be downloaded at: https://www.mdpi.com/article/10.3390/cells15201891/s1, Table S1: Key studies informing the neuroimmune–lipid framework of chronic pruritus.

## Abbreviations

The following abbreviations are used in this manuscript:

AA	arachidonic acid
AD	atopic dermatitis
ATX	autotaxin
C1P	ceramide-1-phosphate
DRG	dorsal root ganglion
GRP	gastrin-releasing peptide
GRPR	gastrin-releasing peptide receptor
IL-31	interleukin-31
LOX	lipoxygenase
LPA	lysophosphatidic acid
LPP	long periodicity phase
NLRP3	NOD-like receptor family pyrin domain-containing 3
PN	prurigo nodularis
S1P	sphingosine-1-phosphate
S1PR3	sphingosine-1-phosphate receptor 3
SC	stratum corneum
SPP	short periodicity phase
TEWL	transepidermal water loss
TNF-α	tumor necrosis factor-α
TRPA1	transient receptor potential ankyrin 1
TRPV1	transient receptor potential vanilloid 1
TSLP	thymic stromal lymphopoietin
