# Supplementary material for: Neuroimmune–Lipid Crosstalk in Chronic Itch: Molecular Mechanisms from Skin Surface to Cerebral Cortex
Source: Cells. 2026 Oct 16;15(20):1891. doi: 10.3390/cells15201891 (PMC13703228; doi:10.3390/cells15201891)
Supplement: Supplementary file 1 [file cells-15-01891-s001.zip › cells-4587072-supplementary.pdf]

Supplementary Table S1. Key studies informing the neuroimmune–lipid framework of chronic pruritus

This table summarizes representative original studies selected for their relevance to the key neuroimmune–lipid mechanisms discussed in this narrative review. Studies are organized by mechanistic level, with emphasis on experimental models, principal findings, and limitations. Reference numbers correspond to the reference list in the main manuscript. This table serves as a narrative evidence map rather than an exhaustive literature list, a systematic-review inclusion log, or a formal study-level quality appraisal.

Study (author, year)	Pathway / context	Design / model	Principal finding discussed	Interpretation / key limitation
Berdyshev E et al. (2018) [19]	Barrier lipids / AD	Human skin lipid analysis	Type 2 cytokine-associated abnormalities in AD skin lipids.	Human disease evidence; direct lipid-to-itch causality not established.
Kanoh H et al. (2019) [22]	Ceramide / barrier	Mouse AD-like model	IFN-γ-associated changes in ceramide fatty-acid composition and barrier function.	Preclinical barrier mechanism; itch-specific effect not established.
Hill RZ et al. (2018) [29]	S1P–S1PR3–TRP	Mouse sensory neurons / behavioral models	S1PR3 mediates itch and pain through distinct TRP-dependent pathways.	Preclinical causal evidence; human itch validation limited.
Langeslag M et al. (2014) [28]	S1P sensory sensitization	Mouse sensory neurons	S1P1–p38 signaling enhances capsaicin-evoked sensory currents.	Neuronal excitability evidence; not direct human itch evidence.
Masuda-Kuroki K et al. (2023) [33]	S1PR2 / skin inflammation	S1PR2-deficient experimental skin model	S. epidermidis modifies allergic contact dermatitis in S1PR2-deficient skin.	Experimental inflammatory context; not direct human itch causality.
MacKnight HP et al. (2019) [35]	C1P–cPLA2α	Experimental wound-healing models	C1P interaction with cytosolic phospholipase A2 coordinates inflammatory lipid signaling during wound repair.	Mechanistic lipid evidence; itch-specific causality unproven.
Jain A et al. (2011) [38]	TRP–eicosanoids	Human keratinocyte experiments	TRP-channel stimulation yields distinct cutaneous eicosanoid release patterns.	In vitro mediator production; integrated in vivo itch loop unproven.
Kremer AE et al. (2010) [41]	ATX–LPA / cholestasis	Human cholestatic disease / mechanistic assays	Identifies LPA as a candidate mediator associated with cholestatic pruritus.	Human association and mechanistic support; direct causal treatment validation limited.
Kittaka H et al. (2017) [43]	LPA5–PLD–TRPA1/TRPV1	Experimental itch models	LPA-evoked itch depends on LPA5-linked phospholipase D and TRP signaling.	Preclinical pruritogenic mechanism; endogenous human cutaneous LPA role unclear.
Fischer C et al. (2024) [44]	LPAR5 / LPAR2	Mouse hydroxychloroquine itch model	LPAR5 and LPAR2 differentially regulate scratching.	Pharmacologically induced mouse itch; disease generalizability unclear.
Oetjen LK et al. (2017) [46]	Cytokine–sensory neurons	Experimental neuronal / inflammatory models	Sensory neuronal cytokine signaling contributes to itch-associated inflammation.	Mechanistic preclinical evidence; not proof of lipid mediation.
Cevikbas F et al. (2014) [47]	IL-31 neuronal itch	Experimental sensory neuron / itch models	Neuronal IL-31 receptor signaling links type 2 inflammation to itch.	Strong neuroimmune mechanism; lipid contribution not established.
Liu B et al. (2016) [48]	IL-33–ST2	Mouse contact allergy model	IL-33/ST2 activates sensory neurons and elicits itch responses.	Preclinical cytokine-neuronal mechanism; not lipid-specific.
Wilson SR et al. (2013) [49]	TSLP–sensory neurons	Experimental sensory neuron / itch models	Keratinocyte-derived TSLP activates sensory neurons to induce itch.	Direct experimental pruriception; lipid metabolic contribution indirect.
Kwatra SG et al. (2023) [52]	IL-31RA / PN	Phase III randomized clinical trial	Nemolizumab reduces pruritus in prurigo nodularis.	Human interventional efficacy; does not establish a lipid-mediated mechanism.
Deng J et al. (2023) [54]	IL-31RA / PN tissue	Human PN samples during treatment	Nemolizumab treatment modifies neuroimmune and epithelial signatures.	Treatment-associated tissue changes; pathway-specific causality uncertain.
Nattkemper LA et al. (2024) [78]	BNP / chronic itch	Human circulating biomarker study	Circulating BNP levels are associated with multiple chronic itch conditions.	Human biomarker association; spinal-to-skin feedback not established.
Meng J et al. (2018) [79]	IL-31–BNP skin–nerve	Experimental neuroimmune models	Links IL-31-associated neuronal responses to peripheral skin signaling.	Experimental pathway; clinical bidirectional feedback not established.

Study (author, year)	Pathway / context	Design / model	Principal finding discussed	Interpretation / key limitation
Mishra SK et al. (2020) [80]	Periostin–integrin	Experimental sensory neuron / itch models	Periostin activates sensory neuronal integrins to promote allergic itch.	Preclinical pruritogenic mechanism; not a direct lipid pathway.
Schmelz M et al. (1997) [60]	Peripheral C-fibers	Human microneurography	Identifies histamine-responsive pruriceptive C-fibers in human skin.	Direct human afferent physiology; not lipid-specific.
Sun YG et al. (2007) [63]	Spinal GRPR signaling	Mouse spinal itch models	Spinal GRPR signaling contributes to itch transmission.	Preclinical spinal causal evidence; direct lipid link not demonstrated.
Kanehisa K et al. (2022) [14]	NPTX2 spinal plasticity	Experimental chronic itch model	NPTX2 facilitates excitatory inputs onto spinal itch-related neurons.	Preclinical spinal plasticity; itch specificity and human relevance uncertain.
Liu X et al. (2023) [67]	NLRP3–IL-1β–GRPR	Mouse chronic itch models	Microglia–neuron signaling amplifies spinal itch through the NLRP3–IL-1β–GRPR axis.	Preclinical spinal neuroimmune mechanism; no direct cutaneous lipid link.
Liu T et al. (2016) [68]	Spinal TLR4	Mouse chronic itch models	TLR4 contributes to chronic scratching, alloknesis and spinal glial activation.	Mouse neuroimmune evidence; lipid-specific activation unestablished.
Shiratori-Hayashi M et al. (2015) [69]	Spinal STAT3 astrocytes	Mouse chronic itch models	STAT3-dependent reactive astrogliosis contributes to chronic itch.	Preclinical downstream mechanism; direct lipid dependence unproven.
Shiratori-Hayashi M et al. (2021) [70]	Astrocyte Ca2+ / STAT3	Mouse chronic itch models	Astrocytic IP3R1/TRPC Ca2+ signals support STAT3 activation and chronic itch.	Preclinical glial pathway; lipid specificity not demonstrated.
Yeo M et al. (2022) [99]	KCC2 / spinal gating	Preclinical itch / neuropathy models	KCC2-enhancing treatment modifies spinal sensory gating in experimental models.	Preclinical; no established clinical antipruritic efficacy.
Schneider G et al. (2008) [74]	Supraspinal itch	Human functional neuroimaging / AD	Histamine-induced itch engages different central responses in AD versus controls.	Human association; no direct lipid-to-cortex causality.
Schut C et al. (2017) [75]	Contagious itch / central processing	Human functional neuroimaging / AD	Contagious itch recruits central processing and motivational networks.	Human functional response; not lipid-specific or proof of chronification.
Li CY et al. (2024) [76]	Brain structure / AD	Human structural neuroimaging	Gray-matter differences correlate with AD severity and inflammatory measures.	Cross-sectional association; direction of causality unknown.
Fishbane S et al. (2020) [72]	Peripheral KOR / CKD-aP	Phase III randomized clinical trial	Difelikefalin provides antipruritic benefit in hemodialysis-associated pruritus.	Human interventional efficacy; not lipid-targeted.
Berdyshev E et al. (2022) [83]	Dupilumab / AD barrier	Human therapeutic study	Dupilumab treatment improves skin barrier function in AD.	Clinical barrier improvement; lipid changes do not prove direct itch mediation.
Deng J et al. (2022) [84]	PN tissue remodeling	Human PN vs AD tissue study	Identifies extracellular-matrix and nerve growth factor dysregulation in PN.	Human tissue association; lipid-specific causal mechanism not shown.
Yu H et al. (2019) [87]	Bile acid–MRGPRX4 / cholestatic itch	Human receptor / experimental itch evidence	Identifies MRGPRX4 as a bile acid-responsive receptor relevant to human itch.	Human-relevant peripheral mechanism; bilirubin contribution clinically uncertain.
Liu L et al. (2021) [90]	S1P / psoriasis	Preclinical and human psoriasis analyses	Examines S1P pathway modulation in psoriasis-like inflammation.	Mixed evidence; itch-specific human causality not established.
Kim BK et al. (2022) [92]	Ceramide / psoriasis	Human psoriasis skin analysis	Reports reduced long-chain ceramide species in psoriasis.	Human lipid association; causal relationship to itch unclear.
Sorokin AV et al. (2018) [93]	Eicosanoids / psoriasis	Human skin and blood lipid profiling	Identifies altered bioactive lipid mediator profiles in psoriasis.	Human lipidomics association; itch-specific mediation unresolved.
Rousel J et al. (2024) [94]	Guselkumab / psoriasis	Human therapeutic skin lipid study	Guselkumab-associated normalization of stratum-corneum ceramide profiles.	Treatment-associated lipid recovery; mediation of itch benefit unproven.
Renkhold L et al. (2024) [95]	Secukinumab / psoriasis	Human therapeutic / skin nerve study	Reports reduced psoriasis-related itch and changes in cutaneous nerve architecture.	Clinical and tissue findings; causal neural mediation unproven.

Study (author, year)	Pathway / context	Design / model	Principal finding discussed	Interpretation / key limitation
Cork MJ et al. (2026) [96]	Dupilumab / barrier	Human AD treatment study	Documents recovery of epithelial barrier function with dupilumab.	Clinical barrier response; direct neuronal/lipid causal pathway unproven.

Abbreviations: AD, atopic dermatitis; ATX, autotaxin; BNP, B-type natriuretic peptide; C1P, ceramide-1-phosphate; CKD-aP, chronic kidney disease–associated pruritus; GRPR, gastrin-releasing peptide receptor; IL, interleukin; KOR, kappa-opioid receptor; LPA, lysophosphatidic acid; PN, prurigo nodularis; PLD, phospholipase D; S1P, sphingosine-1-phosphate; TRP, transient receptor potential; TSLP, thymic stromal lymphopoietin.
